# Diagnostic Insights into Pediatric Pleomorphic Xanthoastrocytoma through DNA Methylation Class and Pathological Diagnosis Analysis

**DOI:** 10.3390/diagnostics13223464

**Published:** 2023-11-17

**Authors:** Murad Alturkustani

**Affiliations:** 1 Department of Pathology, Faculty of Medicine, King Abdulaziz University, Jeddah 21589, Saudi Arabia; alturkustani.murad@gmail.com; Tel.: +966-500936683; 2 Department of Pathology and Laboratory Medicine, Western University, London, ON N6A 5C1, Canada

**Keywords:** pleomorphic xanthoastrocytoma, methylation class, pathological diagnosis, CNS tumor classification

## Abstract

This study adopts an innovative approach to utilize the DNA methylation class (MC) by prioritizing the understanding of discrepancies over traditional direct comparisons with the pathological diagnosis (PD). The aim is to clarify the morphological criteria for pleomorphic xanthoastrocytoma (PXA). Using the Children’s Brain Tumor Network online database, PXA-diagnosed cases were sourced. MCs and *CDKN2A/B* statuses were ascertained using the Heidelberg methylation brain tumor classifier v12.5 (v12.8 for selected cases). Three distinct groups emerged: Group 1 confirmed PXA through both PD and MC (7 cases); Group 2 identified PXA via PD alone (7 cases); and Group 3 diagnosed PXA using MC (5 cases). Key insights from the study include the frequent local infiltration of PXA into gray matter structures, mirroring infiltrative astrocytoma. The MC for PXA stands out for its sensitivity. Cases with a PXA morphological diagnosis diverging from the DNA methylation class warrant attention to newer differential diagnoses such as high-grade astrocytoma with piloid features, pilocytic astrocytoma *NF1*-associated, and NET-PATZ1. Tumors with a MC indicative of PXA but lacking its typical features may, if high-grade, behave as grade 4 gliomas. In contrast, their low-grade counterparts could belong to the PXA morphological continuum. Further research is pivotal for cementing these findings.

## 1. Introduction

Pleomorphic xanthoastrocytoma (PXA) is a glial tumor marked by distinct histopathological features [1,2,3]. It is primarily a superficial solid glial neoplasm of spindle cells, pleomorphic and multinucleated astrocytes, xanthomatous cells, and both eosinophilic and pale granular bodies. The challenge in histological diagnosis arises when the tumor displays non-classical histopathological features, which can overlap with other entities. Ganglioglioma is a frequent differential diagnosis due to shared histological and immunostaining characteristics and molecular alterations. The differentials for PXA with high-grade features are primarily giant cell glioblastoma and epithelioid glioblastoma.

DNA methylation has become a pivotal technique for classifying CNS tumors [4]. As its application for CNS neoplasms has grown, it has also revealed challenges, especially when tumors display histological features resonant of distinct types. Within the methylation class (MC) of PXA, some cases were initially diagnosed as epithelioid glioblastoma, pediatric high-grade glioma, astroblastoma, or embryonal tumors [5]. Conversely, although histologically congruent with PXA, certain tumors nestle under unique methylation subclasses. Discerning the accurate modality between histology and methylation when discrepancies arise is the dilemma. While some experts posit histological diagnosis as the gold standard, with MC serving a confirmatory role [6], another expert group suggests that some MC PXA cases with disparate histological diagnoses might be varied morphological spectrum of PXA [5]. The discordance between histological interpretations and methylation class (MC) diagnosis warrants a thorough reevaluation of the diagnostic avenues. Consequently, understanding the methylation classification for PXA and its diagnostic ramifications becomes crucial.

The study presents a novel application of the methylation class, moving away from direct comparisons with integrated pathological diagnosis. Instead, it delves into understanding discrepancies to refine the morphological criteria for PXA diagnosis. Acknowledging the difficulties in some PXA diagnoses—both histologically and via DNA methylation—this study endeavors to bridge existing gaps and shed light on inherent disparities. By juxtaposing these diagnostic modalities for PXA, the aim is to clarify the diagnostic features, weighing the implications and outcomes of each approach.

## 2. Material and Methods

This study utilizes data from the Children’s Brain Tumor Network (CBTN). The CBTN database compiles anonymized and de-identified patient data from various contributing institutions, ensuring confidentiality. It is important to note that all data in this repository are collected strictly after receiving informed consent from the participants or their legal guardians, in strict compliance with ethical standards and guidelines.

For this specific project, the database was accessed to collate cases, creating a dataset on 11 November 2021, with the last access undertaken on 24 September 2023. This dataset comprised 1029 samples from 898 patients. The data for this study are a subset of the broader ‘Pediatric Brain Tumor Atlas’, accessible by selecting the ‘Open Pediatric Brain Tumor Atlas (OpenPBTA)’ study at “https://pedcbioportal.kidsfirstdrc.org” (last accessed on 24 September 2023).

The primary cohort comprised 613 cases, with diagnoses ranging from PXA to low-grade glioma, high-grade glioma, embryonal tumors, and glioneuronal tumors. The documented diagnosis was designated as the pathological diagnosis (PD), to mimic standard procedural practice for these instances. All 613 cases underwent a methylation profile assessment. Using the Heidelberg methylation brain tumor classifier (v12.5)—from the Molecular Neuropathology website, accessible at (https://www.molecularneuropathology.org/mnp, last accessed on 24 Sepetmeber 2023)—the methylation class (MC) for these tumors was discerned. This classifier elucidates methylation profiling outcomes, including the MC, its corresponding score, and the tumor’s copy number alteration. Given the introduction of the classifier’s newer version (v12.8) during the research duration, the 19 selected cases were also reanalyzed using the new classification.

Eligibility criteria encompassed cases diagnosed as PXA either through MC or PD, coupled with the presence of pertinent scanned images and access to next-generation sequencing (NGS) data. NGS data were obtained from the pathological reports (when available), the online database, and the copy number profile from the methylation profiling report. The latter resource facilitated the assessment of the *CDKN2A/B* status, pinpointing a deletion of *CDKN2A/B* (evidenced by focal shifting of the blue line paired with a sequence or lone red dots, typically falling below a log2 value of 0.4 [6]).

The representative images and the pathological reports were reviewed to summarize each case’s histological features: (1) the growth pattern as solid or infiltrative; (2) the classic histological features of PXA which include the presence of large multinucleated astrocytic cells, spindle cells, xanthomatous (lipidized) cells, and the granular bodies (eosinophilic and pale). The frequency of these features was scored as zero for <1%, one for 1 to less than 5%, two for 5 to 50%, and three for more than 50%; and (3) high-grade features, including significant mitosis, necrosis, and endothelial proliferation. The evaluation of these characteristics was achieved by reviewing the detailed microscopic description in the comprehensive pathology reports and undertaking a meticulous inspection of the available slides. In instances where elements such as xanthoma cells or eosinophilic granular bodies were not present, this fact was clearly articulated in the reports, with slide examinations conducted to validate these statements. When the accessible data proved insufficient for a conclusive assessment, the case in question was omitted, as exemplified by one case.

The inclusion criteria were met in 19 cases, constituting eleven males and eight females, aged between 2 and 20 years, and averaging 11.7 years. Outcomes segregated into three categories: Group 1, where both PD and MC concurred on the PXA diagnosis (7 cases); Group 2, distinguished by PXA diagnosis solely through PD (7 cases); and Group 3, characterized by a PXA determination exclusively via MC (5 cases).

## 3. Results

**Figure 1:**
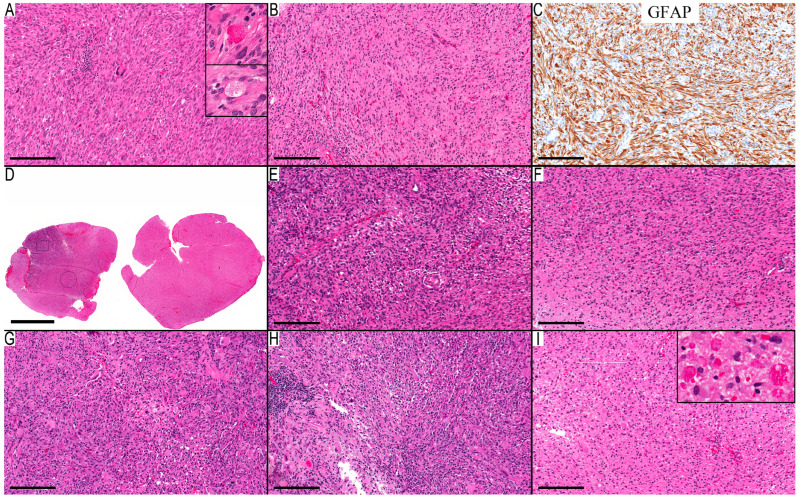
Infiltration in classical PXA cases. (**A**–**C**) Case 1 depicts the classical histological features of PXA: (**A**) a solid cellular region displaying both spindle and pleomorphic cells, accompanied by focal lymphocytic clusters, eosinophilic granular (upper inset), and pale bodies (lower inset); (**B**) an infiltrative component with entrapped neurons; and (**C**) a GFAP immunostain of the neoplastic cells accentuates lengthy, thick processes with pronounced staining. (**D**–**I**) Case 2 with prominent infiltrative component. (**D**–**F**) Initial resection presents features synonymous with infiltrative astrocytoma: (**D**) a broad view of the infiltrative glial neoplasm, highlighting a focal cellular zone; (**E**) the region with heightened cellular activity exhibits oval neoplastic cells against a fibrillary background; and (**F**) an infiltrative component is displaying similar neoplastic cells in less dense cellular regions. (**G**–**I**) Case 2 recurrence with classical PXA features: (**G**) a dense cellular region interspersed with sporadic pleomorphic cells, where the neoplastic cells exhibit large eosinophilic cytoplasm; (**H**) a section with focal lymphocytic infiltration; and (**I**) abundance of eosinophilic granular bodies at the infiltrative edge (higher magnification in the inset). Scale bars: 200 µm (**A**–**C**,**E**–**I**), 2 mm (**D**).

The clinical, histopathological, and the molecular data are summarized in Table 1 for all cases. There were seven cases in the first group, where PD and methylation class confirmed the diagnosis of PXA. These cases uniformly demonstrated the *BRAF* p.V600E mutation and a *CDKN2A/B* deletion. Not all these cases exhibited the quintessential morphological features (Figure 1A–C), particularly concerning xanthomatous neoplastic cells (Table 1). A noteworthy finding is the presence of an infiltrative component in all cases, except for Case 3, where no adjacent gray matter was available for examination. The infiltrative component was the main component in the first resection (at 12 years of age) of Case 2 (Figure 1D–F), where the initial diagnosis was low-grade infiltrative astrocytoma, and the MC was ganglioglioma (0.80). The subsequent recurrence after four years showed classic PXA features (Figure 1G–I) and an MC of PXA with a calibration score of 0.99. This led to reflection upon the initial resection and its accompanying molecular changes, although the morphological features were consistent with infiltrative astrocytoma. However, the *BRAF* p.V600E mutation and *CDKN2A/B* deletion could introduce PXA in the differential diagnosis.

Seven cases were identified in the second group, where PXA diagnosis relied solely on PD. Reevaluation led to alternative diagnoses for four cases: three as ganglioglioma and one as pilocytic astrocytoma. The remaining three cases retained a potential PXA diagnosis (Cases 8 first resection, 10, and 11). Notably, none exhibited the combined presence of *BRAF* p.V600E mutation and *CDKN2A/B* deletion.

**Figure 2:**
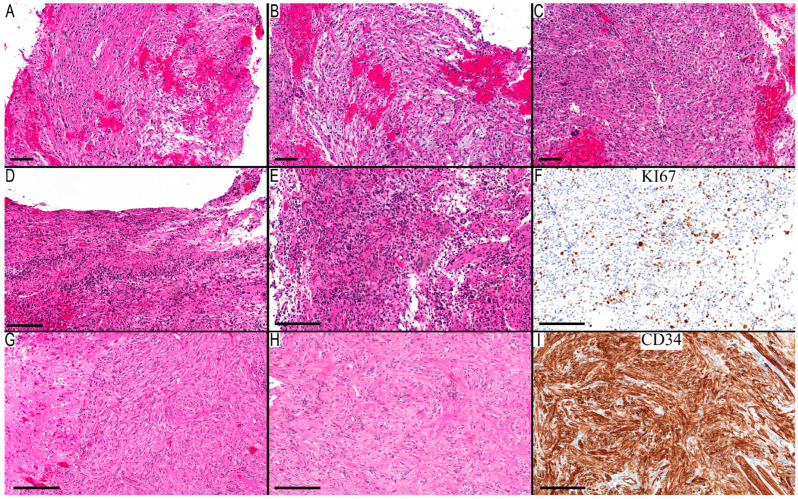
Case of HGAP that progressed from low-grade astrocytoma and a case of NET-PATZ1. (**A**–**F**) Case 8 documents the progression from low-grade astrocytoma to HGAP. (**A**–**C**) the first resection was consistent with low-grade astrocytoma: (**A**) compact area with elongated cells but no convincing Rosenthal fibers; (**B**) microcystic area with myxoid background; and (**C**) occasional giant cells and EGBs. (**D**–**F**) Second resection shows progression to high-grade astrocytoma: (**D**) necrosis with pseudopalisading arrangement; (**E**) cellular areas with round hyperchromatic cells and multinucleated giant cells and EGBs; and (**F**) a Ki67 immunostain shows high proliferative activity. Case 10 (**G**–**I**) is a case of NET-PATZ1 with morphological features of PXA: (**G**) solid circumscribed cellular area with spindle neoplastic cells; (**H**) leptomeningeal infiltration with occasional pleomorphic neoplastic cells; and (**I**) CD34 highlights diffuse staining in the neoplastic cells. Scale bars: 100 µm (**A**–**C**), 200 µm (**D**–**I**).

Case 8’s initial resection was obtained from a basal ganglia–thalamic tumor in a 20-year-old male. This sample displayed a biphasic tumor with a compact area populated by piloid cells and a loose area with a myxoid background (Figure 2A,B). The presence of giant pleomorphic cells (Figure 2C) likely influenced the PD’s classification as PXA. Next-generation sequencing detected an *NF1* Q1822* mutation. Since *NF1*-associated tumors can present with pronounced pleomorphic nuclei, and the MC aligned with PA with a CS of 0.98, the final diagnosis leaned towards pilocytic astrocytoma, *NF1*-associated. However, a PXA diagnosis could not be definitively excluded. Subsequent recurrences presented a diffusely infiltrative astrocytic tumor marked by more significant pleomorphism, necrosis, endothelial proliferation, and notable mitoses (Figure 2D–F). NGS identified the same *NF1* mutation, and an additional *TP53* Y236C mutation appeared in both recurrences. These characteristics aligned with a progression to high-grade astrocytoma. This became the final diagnosis, since the MC indicated high-grade astrocytoma with piloid features (HGAP). Reflecting upon the initial diagnosis of the primary resection, it could have been classified as either pilocytic astrocytoma or PXA, shedding light on the potential evolution of such cases into HGAP.

For Case 10, the morphological features aligned closely with PXA (Figure 2G–I). The MC was identified as NET-PATZ1, and NGS verification of the *MN1-PATZ1* fusion solidified the case’s association with this methylation class. Given the broad morphological spectrum of NET-PATZ1, determining the most fitting final diagnosis for this case presented a challenge. The morphological features of Case 11 highlighted the tumor’s well-defined boundary and a conspicuous presence of epithelioid/rhabdoid cells. There was also a noticeable perivascular arrangement of the neoplastic cells and a lack of high-grade features. While these characteristics can be associated with PXA, they do not exemplify its classical features. Coupled with a “no match” MC, the lack of a *CDKN2A/B* deletion, and the lack of comprehensive NGS data, the PD was categorized as not otherwise specified (NOS).

As for Case 13’s morphological features, it was a well-circumscribed tumor with pleomorphic cells, necrosis, and a *BRAF* p.V600E mutation. While these features align with the PD of PXA, the presence of necrosis and endothelial proliferation with no significant mitosis, the lack of *CDKN2A/B* deletion, and the MC of pilocytic astrocytoma made the final diagnosis a pilocytic astrocytoma. For Cases 9, 12, and 14, the final diagnosis was ganglioglioma, underscoring the challenge of differentiating this from PXA.

The third group had five cases where the PXA diagnosis was derived exclusively from the MC. Their morphological features differed considerably from the classical PXA presentation. These cases can be bifurcated based on their histological grading: high-grade (Cases 15,16 and 19) and low-grade (Cases 17 and 18). All had a *CDKN2A/B* deletion, but only Cases 15 and 19 had the combined *BRAF* p.V600E mutation, with Case 17 revealing a *GTF2I-BRAF* fusion.

**Figure 3:**
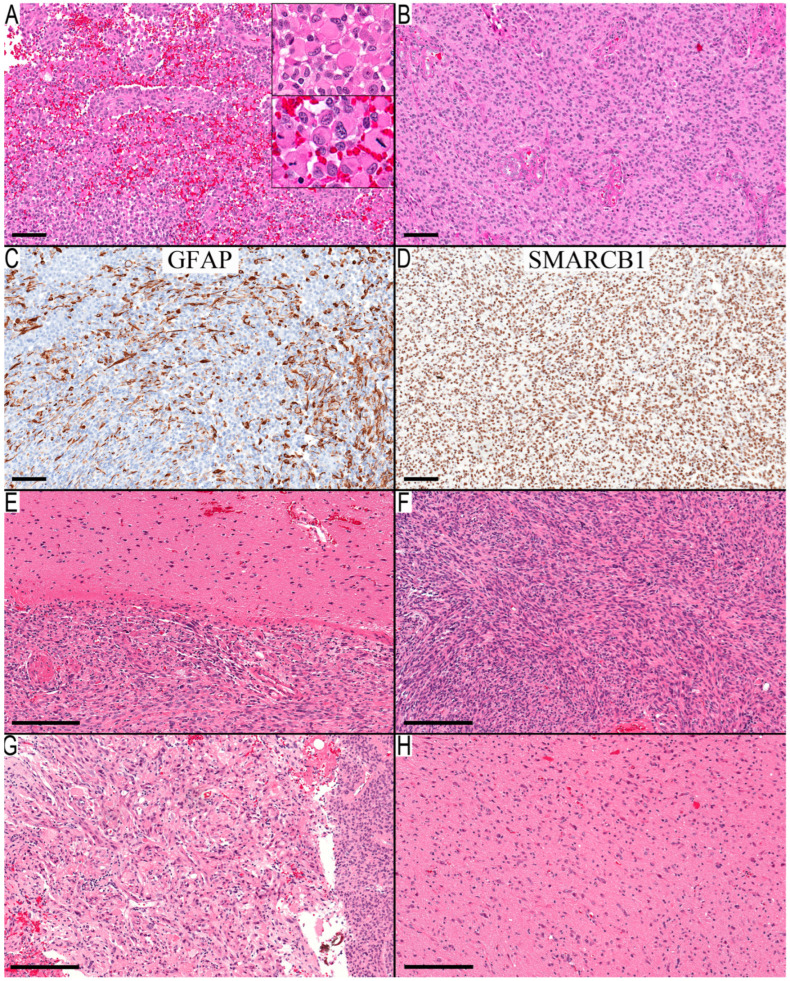
Two cases from the third group with high-grade histological features. (**A**–**D**) Case 15 epithelioid glioblastoma with PXA MC: (**A**) solid sheet of cellular epithelioid/rhabdoid cells (upper inset) with significant mitotic activity (lower inset); (**B**) round cells with fibrillary background and endothelial proliferation; (**C**) GFAP immunostaining shows variable staining ranging from round to elongated cellular processes, but a good proportion of neoplastic cells were immunonegative; and (**D**) SMARCB1 (INI1) immunostain is retained in the neoplastic cells. (**E**–**H**) Case 16 high-grade histology with focal area classic PXA morphological features: (**E**) circumscribed superficial cellular neoplasm; (**F**) high-cellular spindle cell glial neoplasm arranging in interlacing fascicles; (**G**) small focus with many eosinophilic granular and pale bodies; and (**H**) infiltrative component. Scale bars: 100 µm (**A**–**D**), 200 µm (**E**–**H**).

Two distinct infiltration patterns emerged among the two cases with the PD of glioblastoma. Case 15 exhibited diffuse infiltration of epithelioid cells (Figure 3A–D), while Case 16 displayed a more focal infiltration pattern (Figure 3E–H). A specific region in Case 16 bore the classical hallmarks of PXA, a feature absent in Case 15. Case 19, diagnosed initially as an anaplastic glioneuronal tumor with a high-grade histology, was reassessed. Most of its neuronal component consisted of entrapped neurons, not the dysmorphic ganglion cells typical of glioneuronal tumors. Moreover, this case exhibited a combined gain of chromosome 7 and loss of chromosome 10 in its copy number alterations. Consequently, Cases 15 and 19 were diagnosed as diffuse pediatric-type high-grade glioma H3-wildtype and IDH-wildtype. For Case 16, a focal PXA region suggested a possible PXA grade 3. However, the absence of a *BRAF* alteration ultimately classified it as high-grade astrocytoma, not elsewhere classified (NEC). The overall survival for Cases 15 and 19 stood at 16 and 18 months, respectively, while these data were not available for Case 16. This grim prognosis aligns more with a grade 4 astrocytic tumor than a grade 3.

**Figure 4:**
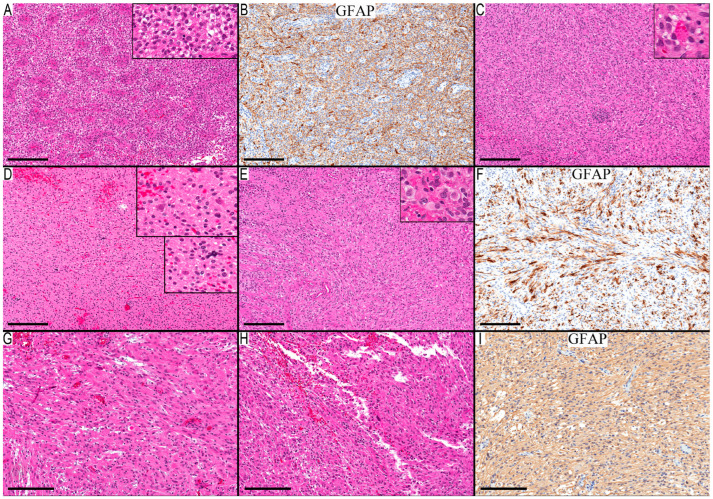
Two cases from the third group with low-grade histological features. (**A**–**F**) Case 17 glial tumor with oligodendroglioma-like and astrocytic-like areas: (**A**) neoplastic cells with round nuclei arranged around multilayered blood vessels (inset show high magnification for the round cells with perinuclear halo); (**B**) GFAP highlights mainly background staining; (**C**) cellular spindle cells with focal lymphocytic aggregates and eosinophilic granular bodies (inset); (**D**) infiltrative component (higher magnification in the upper inset) and microcalcification (higher magnification in the lower inset); (**E**) leptomeningeal involvement with focal rhabdoid/epithelioid cells (inset shows higher magnification for these cells); and (**F**) GFAP is staining cellular processes in the leptomeningeal area. (**G**–**I**) Case 18 low-grade neuroepithelial tumor with histological features of histology of SEGA and *ST13-ROS1* fusion: (**G**) circumscribed tumor composed of neoplastic glial cells with large eosinophilic cytoplasm; (**H**) occasional pleomorphic cells; and (**I**) GFAP shows diffuse faint cytoplasmic staining in the neoplastic cells. Scale bars: 200 µm (**A**–**I**).

Another set comprised two cases with low-grade histology. The morphological traits of Case 17 revealed a component with oligodendroglioma-like histology and spindle cell areas with focal infiltration (Figure 4A–F). Meanwhile, the morphology of the ventricular tumor in Case 18 closely aligned with a subependymal giant cell astrocytoma (SEGA). However, given the absence of clinical evidence for tuberous sclerosis and NGS revealing no mutations in *TSC1* or *TSC2*, the PDs for these two cases were glioma, NEC. The potential for PXA diagnoses remains for both cases, yet this would suggest an expansion of the recognized morphological spectrum of PXA. Given their low-grade histology, the overall progression-free survival for these two cases would not be decisive in differentiating between the potential diagnoses.

## 4. Discussion

This study pioneers a distinct approach to using the methylation class, pivoting away from the conventional practice of juxtaposing it directly against the integrated pathological diagnosis. The core ambition was to unravel and comprehend the discrepancies, allowing a more nuanced recalibration of the morphological criteria used for PXA diagnosis. A salient outcome of this methodological reorientation was elucidating several key morphological criteria: local infiltration into gray matter structures is a consistent finding in PXA, evoking similarities to infiltrative astrocytoma. However, its defining feature is the consistent presence of a solid, non-infiltrative component. The sensitivity of the PXA methylation class is noteworthy. When there is a misalignment between PXA’s morphological diagnosis and its methylation class, it is imperative to factor in newly identified differential diagnosis entities such as HGAP [7], HPAP [8], *NF1*-associated pilocytic astrocytoma [9], and NET-PATZ1 [10]. At a molecular level, PXA’s signature is characterized by MAPK pathway activation, primarily driven by the *BRAF* p.V600E mutation and the *CDKN2A/B* deletion. This signature is pivotal in demarcating PXA from ganglioglioma, a traditionally complex differential. Glial tumors that diverge from typical PXA morphology but marry high-grade features with a PXA methylation class warrant cautious interpretation. These entities can sometimes mimic the behavior of grade 4 gliomas. Some glial tumors, despite deviating from classic PXA characteristics, when displaying low-grade features and matching a PXA methylation class, might be tentatively categorized within the PXA morphological continuum. However, this categorization invites more robust validation through further research.

Integrative diagnoses draw upon a multilayered approach, combining histological and molecular findings to arrive at an integrated pathological diagnosis. However, the precise manner in which the methylation class should be incorporated into this diagnosis remains to be revised. The methylation class operates as an independent methodology, rooted in DNA methylation profiling patterns, and clusters similar entities together [4]. Consequently, the outcome is a juxtaposition between the PD and the tumor’s methylation class (MC), leading to deliberations on which offers a more precise diagnosis. While these criteria continue to evolve, some expert authors assert the superior accuracy of PD for PXA [6].

Conversely, others suggest the potential for broadening the morphological spectrum of PXA to accommodate cases where MC indicates PXA, but PD differs [5]. This study concentrates on elucidating the distinctions between the two diagnostic modalities, sidestepping discussions of their relative superiority to explore avenues for improving diagnostic precision. The MC is contingent upon the tumor’s origin cell and its molecular alterations [4]. Our grasp on why specific tumors are allocated to particular methylation classes remains nascent. Thus, this study emphasizes refining the necessary histological characteristics and molecular outcomes to achieve an accurate PD for PXA.

The first group, as distinguished by a PXA diagnosis confirmed by both PD and MC, offers an avenue to explore the ‘classic’ histological features of PXA. Notably, despite the predominantly solid nature traditionally associated with PXA, all cases in this collection revealed an infiltration into the gray matter when examined. The WHO, while designating PXA as a “circumscribed astrocytic glioma”, admits in its histopathology section that “microscopic invasion at the periphery is common” [1]. The absence of the solid component can tilt the diagnosis towards infiltrative astrocytoma, as evidenced by the first resection in Case 2. Though our sample size is restricted, peripheral infiltration is a recurrent theme in PXA cases. This observation aligns with previous studies indicating that infiltration is a common characteristic in tumors categorized as “circumscribed astrocytic glioma”, such as pilocytic astrocytoma [11] and ganglioglioma [12]. Additionally, the classical features of PXA—pleomorphism and xanthomatous neoplastic cells—are not invariably present. This echoes an earlier study by Giannini et al., which identified xanthic cells in only 66% and giant cells in 92% of 71 PXA cases [13]. Significantly, every case in this category displayed the *BRAF* p.V600E mutation and *CDKN2A/B* deletion. As per the literature, the frequency of these mutations in PXA demonstrates some variability. For instance, *BRAF* p.V600E mutations appeared in approximately 60–66% of PXA cases [14,15,16], and *CDKN2A/B* deletion was documented in 50–70% of cases [5,17]. A recent comprehensive analysis of 67 PXAs marked the deletion in 94% of the specimens and the *BRAF* p.V600E mutation in 76%, with all cases indicating consistent MAPK pathway activation [6]. In conclusion, the combined activation of the MAPK pathway, predominantly through *BRAF* p.V600E, coupled with the *CDKN2A/B* deletion, firmly establishes itself as the molecular signature of PXA.

The second group, defined by an PD of PXA but an MC of a different entity with high CS, provides a window into understanding the pathological features that might intersect with PXA or instances where the MC may not accurately exclude PXA. On reassessment, Case 8 posed a diagnostic conundrum. The morphological and NGS reevaluation of the initial resection could have pointed to either pilocytic astrocytoma with an *NF1* mutation or PXA. Ultimately, the diagnosis harmonized with the MC, a conclusion supported by the follow-up data for this case. The morphological traits of the two subsequent recurrences, combined with molecular alterations and the MC, resonated with HGAP, illustrating the progression from pilocytic astrocytoma to HGAP.

Conversely, Case 10, despite an PD of PXA, presented an MC of NET-PATZ1, emphasizing the complexities and potential ambiguities in establishing a singular diagnosis when both assessment methodologies present valid interpretations. NET-PATZ1, a relatively recent MC, spans a wide morphological range, including cases formerly diagnosed as PXA [10]. Until a more exhaustive dataset emerges, it remains imperative to accentuate both diagnostic possibilities, possibly framing it as glioma, NEC with PXA-reminiscent morphology, and NET-PATZ1 MC.

One of the recurrent challenges in distinguishing PXA lies in its close resemblance to ganglioglioma, attributed to shared histological and immunostaining features coupled with analogous molecular alterations [1]. Distinguishing between these two entities can prove intricate, as evidenced in this study where, in the second group, three out of seven cases diagnosed initially as PXA were later reinterpreted as ganglioglioma. A primary distinguishing feature is the genuine presence of dysplastic or dysmorphic ganglion cells [1] and combined *BRAF* p.V600E and *CDKN2A/B* deletion for PXA. The revised PD of ganglioglioma in these scenarios was grounded by the presence of dysmorphic ganglion cells and the absence of a combined *BRAF* and *CDKN2A/B* molecular signature.

In Case 11, the morphological features do not neatly fall under the PXA archetype but lean more towards a newly identified entity termed high-grade glioma with pleomorphic and pseudopapillary features (HPAP) [8]. Prior to this entity’s recognition, PXA would have been the preferred diagnostic conclusion for such features. The pathologist’s expertise, confidence, and predispositions are crucial in determining whether an PD leans towards a specific diagnosis or is relegated to the NOS/NEC category. For PXA, it might hinge upon the acceptable boundaries of its morphological spectrum, a sentiment echoed in Cases 16–18. The quest for clarity continues, emphasizing the need for comprehensive data on PXA’s acceptable morphological range.

Challenges in discerning the acceptable morphological spectrum become even more pronounced in the third group, where the MC aligns with PXA, but the PD diverges. This poses questions: Do these instances expand PXA’s morphological spectrum, or are they misclassifications by the methylation profiling classifier? A significant limitation of the PXA MC is worth noting—it does not demarcate between grades 2 and 3 [5]. Moreover, the differential diagnosis for the high-grade category encompasses giant cell glioblastoma, epithelioid glioblastoma, and pediatric wild-type glioblastoma, adding complexity to the diagnostic process.

In this study, two cases preliminarily diagnosed as glioblastoma (with a PXA MC and available follow-up data) had notably poor prognoses, enduring for 16 and 18 months. This prognosis aligns more with grade 4 gliomas than with PXA grade 3, hinting at potential classifier misclassification. However, the sample size here is too restrained to draw decisive conclusions regarding the relationship between epithelioid glioblastoma and PXA. Past research has been equivocal: while some early studies suggested they might be variations of a single entity [18], more recent investigations discerned distinct differences [19].

Low-grade feature cases, like cases 17 and 18 in this cohort, infuse the discussion with additional intricacy. Prognostic comparisons are unlikely to offer resolution, given that the marginal difference in low-grade gliomas would necessitate a substantially larger sample size, and such cases are scarcely reported. Hence, the study’s conclusion advocates for adopting the low-grade glioma NOS/NEC categorization while explicitly acknowledging that these could reflect PXA’s broader morphological spectrum.

The study’s retrospective design and limited sample size are its primary limitations. The dependence on detailed pathological reports and representative scanned images for scoring histological features is suboptimal. Some features might not be present in the representative slides or may have been not reported by the primary pathologist. This limitation is particularly pertinent to the scoring of low-incidence features, as their absence cannot be definitively ascertained. However, it does not substantially impact the scoring of high-incidence features. This challenge underscores a broader issue in pathology practice, that of potentially non-representative samples.

A deeper understanding of PXA necessitates more comprehensive, prospective investigations. As the molecular landscape of tumors becomes clearer, periodic updates to the diagnostic criteria for PXA and other similar tumors will be essential. Though progressive, the multi-tiered diagnostic approach endorsed by WHO will need iterative fine-tuning in light of emerging insights. The inclusion of the NOS/NEC designation acknowledges both the gaps in our grasp of the morphological spectrum of these tumors and the limitations intrinsic to the methylation class diagnosis.

In conclusion, this study has innovatively leveraged the methylation class to refine the morphological criteria for PXA diagnosis, moving beyond the traditional approach of comparing with integrated pathological results. Key findings underscore that PXA typically presents local infiltration into gray matter, resembling infiltrative astrocytoma, with an almost invariant solid, non-infiltrative component. Glial tumors that deviate from classic PXA features but align with its methylation class, especially those with high-grade attributes, can exhibit behaviors akin to grade 4 gliomas. Those with low-grade features require further investigation to confirm their place within the PXA spectrum. The sensitivity of the PXA methylation class is underscored, urging caution when confronting a differing methylation class and prompting consideration of new differential diagnoses like HGAP, HPAP, pilocytic astrocytoma *NF1*-associated, and NET-PATZ1. Crucially, PXA’s molecular signature, characterized by MAPK pathway activation predominantly via *BRAF V600E* and the *CDKN2A/B* deletion, provides a robust tool to differentiate it from close differentials such as ganglioglioma.

## Figures and Tables

**Table 1:** Clinical and pathological data.

Case Number	Pathological Diagnosis	MC v12.8 (CS)	Final Diagnosis	Age in Years	Sex	Location	Circumscription	Pleomorphism	Spindle Cells	Xanthoma Cells	Bodies (EGBs/Pale)	Epithelioid/Rhabdoid Cells	Lymphocytes	Mitotic Activity	Necrosis	Endothelial Proliferation	*BRAF* V600E	*CDKN2A/B* Deletion	p*TERT*	PFS (Months)	OS
C1	PXA, G2	PXA (0.99)	PXA	10	M	PL	CFI	0	1	0	1	0	2	NS	No	No	Yes	Yes	No	57	57 (A)
C2	AS	GG (0.80)	AS	12	F	TL	CFI	1	2	0	0	0	1	NS	No	No	Yes	Yes	No	49	91 (A)
C2-R	PXA, G2	PXA (0.99)	PXA	16	F	TL	CFI	2	1	0	2	0	1	NS	No	No	Yes	Yes	No		
C3	PXA, G2	PXA (0.99)	PXA	9	F	FL	C **	1	0	0	1	0	0	NS	No	No	Yes	Yes	No	74	74 (A)
C4	PXA, G2	PXA (0.99)	PXA	14	F	TL	CFI	3	0	0	1	1	2	NS	No	No	Yes	Yes	No	NA	NA
C5	PXA, G2	PXA (0.99)	PXA	13	M	TL	CFI	2	1	1	1	1	2	NS	No	No	Yes	Yes	No	67	67 (A)
C6	PXA, G2	PXA (0.99)	PXA	14	F	FL	CFI	2	1	1	1	0	2	NS	No	No	Yes	Yes	No	9	44 (A)
C7	PXA, G2	PXA (0.99)	PXA	14	M	TL	CFI	3	0	1	1	0	2	NS	No	No	Yes	Yes	No	3	51 (A)
C8	PXA, G2	PA (0.98)	PA, NF1 *	20	M	BGT	C **	2	1	0	1	0	1	NS	No	No	No	Yes	Yes	31	108 (D)
C8-R	PXA, G3	HGAP (0.97)	HGAP	26	M	BGT	CDI	2	0	0	1	1	1	S	Yes	Yes	No	Yes	Yes		
C8-R	PXA, G3	HGAP (0.89)	HGAP	26	M	BGT	CDI	2	1	0	0	0	0	S	Yes	Yes	No	Yes	Yes		
C9	PXA, G2	GG (045)	GG	16	F	PL	CFI	0	0	0	1	0	1	NS	No	No	NA	No	NA	50	50 (A)
C10	PXA, G2	NET-PATZ1 (0.98)	NET-PATZ1 *	3	M	FL	CFI	0	1	0	0	0	1	NS	No	No	No	No	No	15	15 (A)
C11	PXA, G2	PA, midline (0.49)	GL, NOS *	17	M	FL	C	1	1	0	1	3	1	NS	No	Yes	NA	No	NA	NA	NA
C12	PXA, G2	CT (0.86)	GG	16	M	TL	CDI	0	3	0	0	0	1	NS	No	No	Yes	No	No	12	12 (A)
C13	PXA, G2	PA, HE (0.98)	PA	16	M	TL	C	2	0	0	0	0	1	NS	Yes	Yes	Yes	No	No	NA	NA
C14	PXA, G2	GG (0.81)	GG	13	F	PL	CFI	1	1	0	0	0	1	NS	No	No	Yes	No	No	NA	NA
C15	GBM	PXA (0.98)	pHGG	2	M	TL	CDI	1	0	0	0	3	0	S	Yes	Yes	Yes	Yes	No	16	16 (D)
C16	GBM	PXA (0.96)	GL-HG, NEC *	13	M	FL	CFI	1	2	1	2	0	1	S	Yes	No	No	Yes	No	NA	NA
C17	GL, NEC	PXA (0.90)	GL, NEC *	5	M	TL	CFI	1	1	0	1	1	1	S	No	Yes	No	Yes	No	56	56 (A)
C18	GL, NEC	PXA (0.92)	GL, NEC *	6	F	LV	C **	2	2	0	0	3	1	NS	No	No	No	Yes	No	32	32 (A)
C19	GNT-HG	PXA (0.99)	pHGG	9	F	TL	CFI	0	0	0	0	1	1	S	No	Yes	Yes	Yes	No	18	18 (D)

* The possibility of PXA cannot be excluded; ** no adjacent gray matter available to examine infiltration; frequency of observations: zero ≤ 1%, one = 1–<5%, two = 5–50%, three ≥ 50%; abbreviations: A: alive; BGT: basal ganglia and thalamus; C: circumscribed; CDI: circumscribed and diffusely infiltrative; CFI: circumscribed and focally infiltrative; CS: calibration score; CT: control tissue; D: dead; EGB: eosinophilic granular bodies; F: female; FL: frontal lobe; G: grade; GBM: glioblastoma; GG: ganglioglioma; GL: glioma; GNT: glioneuronal tumor; HE: hemispheric; HG: high-grade; HGAP: high-grade astrocytoma with piloid features; LV: lateral ventricle; M: male; MC: methylation class; NEC: not elsewhere classified; NET: neuroepithelial tumor; *NF1*: *NF1*-associated; NOS: not otherwise specified; NS: not significant (<2.5 mitoses/mm^2^); OS: overall survival; PFS: progression-free survival; pHGG: diffuse pediatric-type high-grade glioma H3-wildtype and IDH-wildtype; PL: parietal lobe; R: recurrent; S: significant (>2.5 mitoses/mm^2^); TL: temporal lobe.

## Data Availability

All relevant data files are available from the corresponding author (MA) upon request.
